# Intrauterine Extremity Gangrene and Cerebral Infarction at Term: A Case Report

**DOI:** 10.1155/2011/363517

**Published:** 2011-12-20

**Authors:** M. Tanvig, J. S. Jørgensen, M. Nybo, G. Zachariassen

**Affiliations:** ^1^Department of Gynecology and Obstetrics, Odense University Hospital, 5000 Odense, Denmark; ^2^Department of Clinical Biochemistry and Pharmacology, Odense University Hospital, 5000 Odense, Denmark; ^3^Hans Christian Andersen Children's Hospital, Odense University Hospital, 5000 Odense, Denmark

## Abstract

Intrauterine extremity gangrene in combination with cerebral infarction is a serious and rare event. We present a case with a healthy mother who gave birth to a child with this condition. At term, the mother presented at the antenatal clinic with decreased fetal movements. Cardiotocography (CTG) showed signs of fetal distress and a caesarean section was performed. The left arm of the newborn was found gangrenous. Amputation of the arm was necessary and the child was subsequently treated with anticoagulant therapy due to thrombosis and cerebral infarction in the left hemisphere found by magnetic resonance imaging (MRI). At one year of age the boy was doing well and had prosthesis as a left arm. He had no signs of further complications. Despite thorough examination of the parents and the child, the reason for the thrombosis is still unknown.

## 1. Introduction

Intrauterine thrombosis with extremity gangrene presenting at birth is a rare event with a limited number of cases described in the literature [1–14]. By far, extremity gangrene in combination with cerebral infarction is even rarer. We present a case of intrauterine fetal limb gangrene in combination with cerebral infarction.

Intrauterine thrombosis should be distinguished from neonatal thrombosis, which occurs after birth. Neonatal thrombosis is often caused by catherization of the umbilical artery in a sick neonate [1], or seen as complication to sepsis or coagulation disorders [15].

The pathogenesis of intrauterine gangrene can be divided into intrauterine compression or thromboembolic phenomena [16]. The compression is generally caused by uterine anomalies, fetal malpresentation with limb prolapse, oligohydramnios, amniotic bands, or umbilical cord entanglement [4, 14, 17–20]. 

Intrauterine fetal ischemia caused by thrombosis or emboli has been linked to maternal diabetes [1, 8, 10, 14], preterm delivery [2], dehydration, polycythaemia, and twin-to-twin transfusion syndrome [1, 21].

Neonates are in a transient thrombophilic state with low activity of protein C, protein S, antithrombin, plasminogen, and tissue plasminogen activator [1, 22–24]. The risk of thrombosis is even higher if any of the above conditions are present. Recently, three case reports have linked intrauterine arterial thrombosis with methylenetetrahydrofolate reductase (MTHFR) mutations and factor V Leiden mutation [3, 6, 7]. This connection needs further investigation, though.

Also, emboli can originate in the placenta and pass through the foramen ovale to lodge in the arterial system, predominantly causing upper-limb necrosis [1, 5, 14].

However, in many of the reported cases, the precise pathogenesis of the thrombosis has not been found.

Whatever the cause, intrauterine thrombosis can have devastating consequences. It requires thorough attention and collaboration between obstetricians, neonatologists, orthopedics, and plastic surgeons. The treatment varies from case to case. Initially, it is important to save viable structures. In many cases, though, amputation is necessary. Other aspects of treatment can be thrombolysis and anticoagulant therapy.

## 2. Case Presentation

A 31-year-old woman with a prior normal pregnancy and delivery presented at term to the antenatal clinic as she had felt decreased fetal movements for the past three days. During the same period, she was feeling nauseous and unwell, but she had no fever. Until then, the pregnancy had been uneventful. She had a pregestational BMI of 29 kg/m^2^. There was no history of maternal hypertension, infection, or hypercoagulopathy, and she had a normal oral glucose tolerance test (2 hours value: 5,6 mmol/L, reference value (ref.): ≤9.0 mmol/L). Ultrasound screenings at 12 + 5 weeks and 20 + 3 weeks of gestation were normal.

At arrival the CTG was pathological, with a silent pattern, a mild tachycardia, and no accelerations. The mother had a blood pressure of 140/110, maternal pulse rate was 100 beats per minute, and her temperature was normal. An emergency caesarean section (EMCS) was performed. Amniotic fluid volume and smell were normal, but the amniotic fluid was unclear. Both macroscopic and microscopic examinations of the placenta were normal.

**Figure 1:**
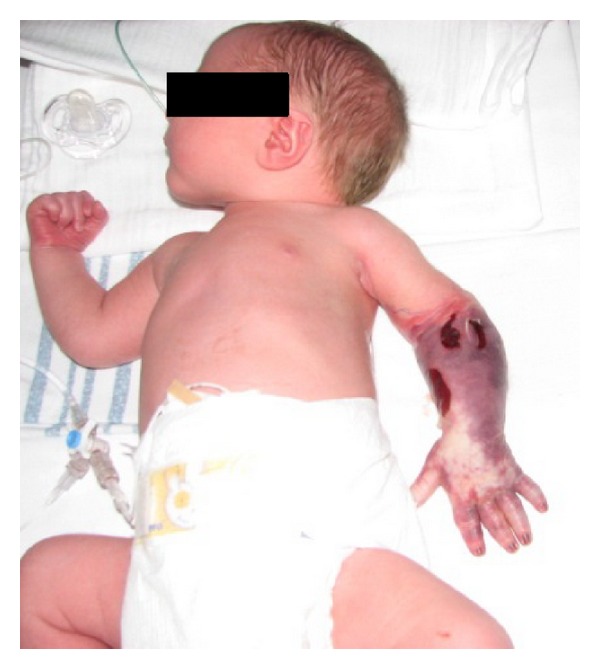


The newborn boy weighed 3085 g, the length was 51 cm, and head circumference was 35 cm. Apgar scores were 5/1, 8/5, and 10/10. Umbilical cord blood gas values were arterial pH 7,19 (ref. >7,10) and base excess −7,0 mmol/L (ref. >−10). Cyanosis of the left arm from the elbow and down was immediately noted (Figure 1). The epidermis was spontaneously cracking and fell off merely by touching. There was limited movement of the arm. The boy was shortly ventilated and then due to tachypnea and grunting treated with nasal continuous positive airway pressure (n-CPAP). Blood samples right after birth yielded the following results: lactate 12 mmol/L (ref. 0,5–2,1), pH 7.23 (ref. 7,35–7,45), and Base excess −10 (ref. >−3,0). Coagulation factors, haemoglobin, and platelet count were normal. The values were coagulation factors II, VII, X: 0,7 IU/L (ref. 0,7–1,3), coagulation factors I, II, V, VII, XII: 35 s (ref. 27–40 s), haemoglobin 10,8 mmol/L (ref. 7,0–11,5), and platelets 152*10^9^/L (ref. 120–400).

Closer examination of the lower part of the upper-left extremity provided evidence of gangrene, and the condition was spreading proximally within a few hours. No pulse was detected in arteria brachialis, radialis, or ulnaris by Doppler ultrasound. A normal signal was detected in arteria axillaris. Within hours after birth, amputation was inevitable and was performed at a midhumerus level.

Before amputation was carried out, the boy developed 3 short episodes of seizures in his upper-right extremity. No seizures were observed after amputation. Magnetic resonance imaging (MRI) of the cerebrum four days after birth showed a thrombosis of the vena intracranial and cerebral infarction in the left parietal lobe. Concomitantly, fibrin D-dimer rose from 2.14 to 13.3 mg/L (ref. <0,5). Treatment with prophylactic heparin injections (Fragmin) (100 IE/kg/day) was subsequently initiated. This treatment continued until four months of age. Additional cerebral MRI showed no signs of venous thrombosis. However, a certain loss of cortical tissue on the left side was evident.

Both parents were examined in order to rule out thrombophilia as a potential culprit, and screening included the following factors: factor V Leiden mutation, prothrombin mutation (G20210A), activated protein C resistance, free protein S concentration, total protein S concentration, total protein C concentration, antithrombin concentration, antiphospholipid antibodies (including lupus anticoagulans, *β*2-glycoprotein 1 antibodies, and cardiolipin antibodies), and homocysteine (to rule out MTHFR mutations). Platelet number and size (MPV) were investigated. All tests turned out normal; hence, thrombophilia due to biochemical factors was ruled out as the reason for this event. For further information on the results of the tests done on both parents, see Table 1.

**Figure 2:**
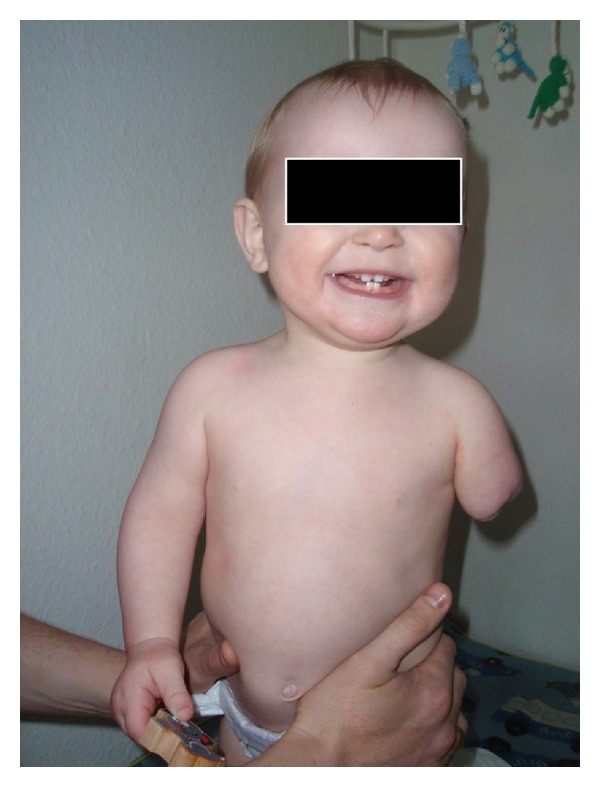


**Figure 3:**
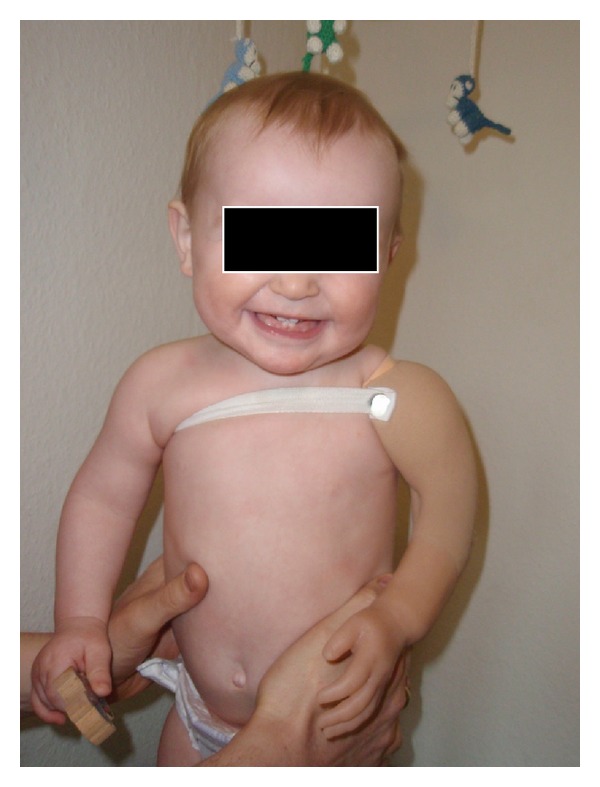


The boy got his first prosthesis at an age of six months. Eleven-month old, he was seen at the pediatric outpatient clinic: He was not yet able to crawl, but was moving forward using his 3 normal limbs. He could stand up with a little support and did not show any signs of spasticity. The new prosthesis (Figures 2 and 3) was worn at all times, except when he was asleep. He was recognizing the left artificial limb and was using his right arm to move it.

## 3. Discussion

Various pathological conditions can cause congenital gangrene. The most common are amniotic bands, umbilical cord compression, oligohydramnios, intrauterine thrombosis, and placental emboli [5]. In the present case, gangrene of the left arm was probably followed by cerebral infarction in the left hemisphere. So far, the combination of intrauterine extremity ischemia and cerebral infarction has only been described in a handful of cases in the literature [9, 11–13]. This rare event should be approached with great care. The case illustrates the necessity of considering the possibility of cerebral involvement when a neonate presents with extremity ischemia. The threshold of performing a cerebral MRI should be low.

Since an inherited prothrombotic state of the mother, or in some case the father, increases the risk of thrombosis in the neonate [25], it is advisable to rule out deficiencies and/or thrombophilic factors in the coagulation system. It is important to test the parents for various factors; see Table 2 for further information. Of course, the child should also have coagulation factors, haemoglobin, and platelet count evaluated. In our case, none of the mentioned parameters were abnormal. In cases of limb ischemia, a conservative approach is often preferable. In our case, however, the gangrene was rapidly spreading, and amputation was inevitable. Anticoagulation therapy was started immediately after the cerebral thrombosis was confirmed.

The mother's sensation of decreased fetal movements was the initial event that led to the EMCS. The CTG provided evidence of fetal distress, indicating an alarming intrauterine condition of the fetus. Decreased fetal movements can be signs of fetal compromise such as intrauterine growth restriction (IUGR), chronic asphyxia, or fetal death (IUFD) [26]. However, in the present case, this was not the situation. The mother was feeling nauseous for a couple of days before the delivery, but she had no other signs of disease or infection.

The direct cause of the thrombosis was never found. The mother was healthy, had no glucose intolerance or hypertension. No signs of infection were found, and the placenta and the uterus were normal with no amniotic bands. Thrombophilic factors were ruled out as a cause of the condition. It has previously been described that emboli from the placenta can pass through the foramen ovale and lodge in the arterial system, usually causing upper-limb necrosis [1, 5, 14]. One could speculate that this was the reason for both the upper-limb involvement and the cerebral infarction, even though we did not find any evidence of thromboses in the placenta.

Now the boy is one year old and he is doing well. He has a well-functioning prosthesis and shows no signs of sequelae from the cerebral infarction. Furthermore, he has no signs of further complications.

## Figures and Tables

**Table 1:** 

Test	Mother	Father	Reference value
Factor V Leiden mutation	Negative	Negative	Negative
Prothrombin mutation (G20210A)	Negative	Negative	Negative
Free protein S concentration	0,7∗10^3^ IU/L	1,02∗10^3^ IU/L	0,5–1,3∗10^3^ IU/L
Total protein S concentration	0,64∗10^3^ IU/L	0,98∗10^3^ IU/L	0,5–1,3∗10^3^ IU/L
Activated protein C resistance	2,9	2,9	>2,4
Total protein C concentration	1,36∗10^3^ IU/L	1,03∗10^3^ IU/L	0,67–1,4∗10^3^ IU/L
Antithrombin concentration	0,91 ∗10^3^ IU/L	0,95∗10^3^ IU/L	0,8–1,2∗10^3^ IU/L
Lupus anticoagulants	0	0	0
*β*2-glycoprotein 1 antibodies IgG	3∗10^3^ IU/L	3∗10^3^ IU/L	<10∗10^3^ IU/L
*β*2-glycoprotein 1 antibodies IgM	2∗10^3^ IU/L	2∗10^3^ IU/L	<6∗10^3^ IU/L
Cardiolipin antibodies IgG	<1∗10^3^ IU/L	2∗10^3^ IU/L	<10∗10^3^ IU/L
Cardiolipin antibodies IgM	<1∗10^3^ IU/L	2∗10^3^ IU/L	<10∗10^3^ IU/L
Homocysteine	7,4 *μ*mol/L	6,94 *μ*mol/L	4,5–12 *μ*mol/L
Platelets	217∗10^9^/L	209∗10^9^/L	120–400∗10^9^/L

**Table 2:** Parental screening for thrombophilia.

Factor V Leiden mutation	
Prothrombin mutation (G20210A)	
Free protein S concentration	
Total protein S concentration	
Activated protein C resistance	
Total protein C concentration	
Antithrombin concentration	
Antiphospholipid antibodies	
Homocysteine	
